# Author Correction: Profiling crRNA architectures for enhanced Cas12 biosensing

**DOI:** 10.1038/s42003-025-08645-0

**Published:** 2025-08-12

**Authors:** Elizabeth Toyin Ajibode, Alexandra R. Bender, Kevin Yehl

**Affiliations:** Department of Chemistry and Biochemistry, Miami University, Oxford, Oxford, OH USA

**Keywords:** Biosensors, Enzyme mechanisms

Correction to: *Communications Biology* 10.1038/s42003-025-08356-6, published online 21 June 2025

In the version of the article initially published, the magnitude of V_max_ values shown in Fig. 4d–f was incorrect due to a calculation error; the *y*-axis ranges and reported V_max_ values are now amended in the HTML and PDF versions of the article. The authors thank Juan Santiago and Alexandre Avaro for identifying the error.

