# Supplementary material for: Profiling crRNA architectures for enhanced Cas12 biosensing
Source: Commun Biol. 2025 Jun 21;8:947. doi: 10.1038/s42003-025-08356-6 (PMC12182579; doi:10.1038/s42003-025-08356-6)
Supplement: Supplementary file 1 — Supplemental Material [file 42003_2025_8356_MOESM1_ESM.pdf]

Supplementary Information

Profiling crRNA Architectures for Enhanced Cas12 Biosensing

Elizabeth T. Ajibode[1], Alexandra R. Bender[1], Kevin Yehl[1]*

[1]*Department of Chemistry and Biochemistry, Miami University, Oxford, Ohio, United States*

*Email: yehlk@miamioh.edu; ajibodet@miamioh.edu

Contents

Supplementary Table 1. List of primers and gRNA/DNA sequences

Supplementary Figure 1. Schematic showing transcription crRNAs

Supplementary Figure 2. Trans-cleavage activity of DNA reporter substrates

Supplementary Figure 3. Denaturation gel to confirm dimerization of crRNA-30

Supplementary Figure 4. Comparison of Cas12 and crRNA activity from different experiments

Supplementary Figure 5. LoD studies for LbCas12a

Supplementary Figure 6. Performance of Cas12-crRNA-20 and target DNA in a mixed pool of non-target DNA

Supplementary Figure 7. SNP studies for Cas12

Supplementary Figure 8. Heat map for Cas12 SNP studies

Supplementary Figure 9. Comparison of Cas12 activity with linear and plasmid DNA

Supplementary Figure 10. Investigating background Activity with no DNA

Supplementary Figure 11. Investigating background Activity with different DNAs

Supplementary Figure 12. Uncropped gel image from Figure 6a

Supplementary Figure 13. Predicted bivalent crRNA secondary structures

Supplementary Table 1. List of primers and gRNA sequences

Oligonucleotides	Sequences (5′ - 3′)
Monovalent<br>crRNA-15<br>crRNA-20<br>crRNA-25<br>crRNA-30<br>crRNA-35<br>crRNA-40	 CCCCCAGCGCUUCAG<br>CCCCCAGCGCUUCAGCGUUC<br>CCCCCAGCGCUUCAGCGUUCUUCGG<br>CCCCCAGCGCUUCAGCGUUCUUCGGAAUGU<br>CCCCCAGCGCUUCAGCGUUCUUCGGAAUGUCGCGC<br>CCCCCAGCGCUUCAGCGUUCUUCGGAAUGUCGCGCAUUGG
Bivalent crRNA (L0)<br><br><br>Bivalent crRNA (L05)<br><br><br><br>Bivalent crRNA (L10)<br><br><br><br>Bivalent crRNA (L15)<br><br><br><br>Bivalent crRNA (L20)	GGUAAUUUCUACUAAGUGUAGAUCCCCCAGCGCUUCAGCGUUCCCUAAGG<br>CACUAUAUGCCAAGUUUUAGAGCUAGAAAUAGCAAGUUAAAAUAAGGCUA<br>GUCCGUUAUCAACUUGAAAAAGUGGCACCGAGUCGGUGCUUU<br>GGUAAUUUCUACUAAGUGUAGAUCCCCCAGCGCUUCAGCGUUCCCUAAGG<br>CACUAUAUGCCAAACCGA GUUUUAGAGCUAGAAAUAGCAAGUUAA<br>AAUAAGGCUAGUCCGUUAUCAACUUGAAAAAGUGGCACCGAGUCGGUGC<br>UUU<br>GGUAAUUUCUACUAAGUGUAGAUCCCCCAGCGCUUCAGCGUUCCCUAAGG<br>CACUAUAUGCCAAACCGAAGCCGGUUUUAGAGCUAGAAAUAGCAAGUUAA<br>AAUAAGGCUAGUCCGUUAUCAACUUGAAAAAGUGGCACCGAGUCGGUGC<br>UUU<br>GGUAAUUUCUACUAAGUGUAGAUCCCCCAGCGCUUCAGCGUUCCCUAAGG<br>CACUAUAUGCCAAACCGAAGCCGCUAGCGUUUUAGAGCUAGAAAUAGCAA<br>GUUAAAAUAAGGCUAGUCCGUUAUCAACUUGAAAAAGUGGCACCGAGUC<br>GGUGCUUU<br>GGUAAUUUCUACUAAGUGUAGAUCCCCCAGCGCUUCAGCGUUCCCUAAGG<br>CACUAUAUGCCAAACCGAAGCCGCUAGCGCUACGUUUUAGAGCUAGAAAU<br>AGCAAGUUAAAAUAAGGCUAGUCCGUUAUCAACUUGAAAAAGUGGCACC<br>GAGUCGGUGCUUU
Bivalent Target DNA (same as gblock wt)	CATTGGCCGCAAATTGCACAATTTGCCCCCAGCGCTTCAGCGTTCTTCGGAAT<br>GTCGCGCATTGGACCGAAGCCGCTAGCGCTACCGGCCAGCCCCGACA
pUC19-SARS-CoV-2 N-gene plasmid	GCGCCCAATACGCAAACCGCCTCTCCCCGCGCGTTGGCCGATTCATTAATG<br>CAGCTGGCACGACAGGTTTCCCGACTGGAAAGCGGGCAGTGAGCGCAACG<br>CAATTAATGTGAGTTAGCTCACTCATTAGGCACCCCAGGCTTTACACTTTAT<br>GCTTCCGGCTCGTATGTTGTGTGGAATTGTGAGCGGATAACAATTTCACACA<br>GGAAACAGCTATGACCATTGGCCGCAAATTGCACAATTTGCCCCCAGCGCT

	TCAGCGTTCTTCGGAATGTCGCGCATTGGCATGGAAGTCACACCTTCGGGA<br>ACGTTAAGCCAGCCCCGACACCCGCCAACACCCGCTGACGCGCCCTGACGG<br>GCTTGTCTGCTCCCGGCATCCGCTTACAGACAAGCTGTGACCGTCTCCGGGA<br>GCTGCATGTGTCAGAGGTTTTCACCGTCATCACCGAAACGCGCGAGACGAAA<br>GGGCCTCGTGATACGCCTATTTTTATAGGTTAATGTCATGATAATAATGGTTT<br>CTTAGACGTCAGGTGGCACTTTTCGGGGAAATGTGCGCGGAACCCCTATTTGT<br>TTATTTTTCTAAATACATTCAAATATGTATCCGCTCATGAGACAATAACCCTG<br>ATAAATGCTTCAATAATATTGAAAAAGGAAGAGTATGAGTATTCAACATTTC<br>CGTGTCGCCCTTATTCCCTTTTTTGCGGCATTTTGCCTTCCTGTTTTTGCTCAC<br>CCAGAAACGCTGGTGAAAGTAAAAGATGCTGAAGATCAGTTGGGTGCACGA<br>GTGGGTTACATCGAACTGGATCTCAACAGCGGTAAGATCCTTGAGAGTTTTC<br>GCCCCGAAGAACGTTTTCCAATGATGAGCACTTTTAAAGTTCTGCTATGTGG<br>CGCGGTATTATCCCGTATTGACGCCGGGCAAGAGCAACTCGGTCGCCGCATA<br>CACTATTCTCAGAATGACTTGGTTGAGTACTCACCAGTCACAGAAAAGCATC<br>TTACGGATGGCATGACAGTAAGAGAATTATGCAGTGCTGCCATAACCATGAG<br>TGATAACACTGCGGCCAACTTACTTCTGACAACGATCGGAGGACCGAAGGAG<br>CTAACCGCTTTTTTGCACAACATGGGGGATCATGTAACTCGCCTTGATCGTTG<br>GGAACCGGAGCTGAATGAAGCCATACCAAACGACGAGCGTGACACCACGAT<br>GCCTGTAGCAATGGCAACAACGTTGCGCAAACTATTAACTGGCGAACTACTT<br>ACTCTAGCTTCCCGGCAACAATTAATAGACTGGATGGAGGCGGATAAAGTTG<br>CAGGACCACTTCTGCGCTCGGCCCTTCCGGCTGGCTGGTTTATTGCTGATAAA<br>TCTGGAGCCGGTGAGCGTGGGTCTCGCGGTATCATTGCAGCACTGGGGCCAG<br>ATGGTAAGCCCTCCCGTATCGTAGTTATCTACACGACGGGGAGTCAGGCAAC<br>TATGGATGAACGAAATAGACAGATCGCTGAGATAGGTGCCTCACTGATTAAG<br>CATTGGTAACTGTCAGACCAAGTTTACTCATATATACTTTAGATTGATTTAAA<br>ACTTCATTTTTAATTTAAAAGGATCTAGGTGAAGATCCTTTTTGATAATCTCAT<br>GACCAAAATCCCTTAACGTGAGTTTTCGTTCCACTGAGCGTCAGACCCCGTA<br>GAAAAGATCAAAGGATCTTCTTGAGATCCTTTTTTTCTGCGCGTAATCTGCT<br>GCTTGCAAACAAAAAAACCACCGCTACCAGCGGTGGTTTGTTTGCCGGATC<br>AAGAGCTACCAACTCTTTTTCCGAAGGTAACTGGCTTCAGCAGAGCGCAGA<br>TACCAAATACTGTTCTTCTAGTGTAGCCGTAGTTAGGCCACCACTTCAAGA<br>ACTCTGTAGCACCGCCTACATACCTCGCTCTGCTAATCCTGTTACCAGTGG<br>CTGCTGCCAGTGGCGATAAGTCGTGTCTTACCGGGTTGGACTCAAGACGATA<br>GTTACCGGATAAGGCGCAGCGGTCGGGCTGAACGGGGGGTTCGTGCACACA<br>GCCCAGCTTGGAGCGAACGACCTACACCGAACTGAGATACCTACAGCGTGA<br>GCTATGAGAAAGCGCCACGCTTCCCGAAGGGAGAAGGCGGACAGGTATCC<br>GGTAAGCGGCAGGGTCGGAACAGGAGAGCGCACGAGGGAGCTTCCAGGGG<br>GAAACGCCTGGTATCTTTATAGTCCTGTCGGGTTTCGCCACCTCTGACTTGA

	GCGTCGATTTTTGTGATGCTCGTCAGGGGGGCGGAGCCTATGGAAAAACGC CAGCAACGCGGCCTTTTTACGGTTCCTGGCCTTTTGCTGGCCTTTTGCTCACA TGTTCTTTCCTGCGTTATCCCCTGATTCTGTGGATAACCGTATTACCGCCTTT GAGTGAGCTGATACCGCTCGCCGCAGCCGAACGACCGAGCGCAGCGAGTCA GTGAGCGAGGAAGCGGAAGA
Cas12 linear reporter substrate	5′- /56-FAM/TTT TTA TTT TT/3IABkFQ/ -3′
Hairpin substrate	5′- /56-FAM/TGGTTATTCCA/3IABkFQ/ -3′
Linear DNA	GCAAATTGCACAATTTGCCCCCAGCGCTTCAGCGTTCTTCGGAATGTCGCGCATTGGCA

*Red sequences represent linker. The sequence highlighted in yellow represents the SARS-CoV-2 N gene sequence (truncated). Underlined sequences represent the PAM sequence.

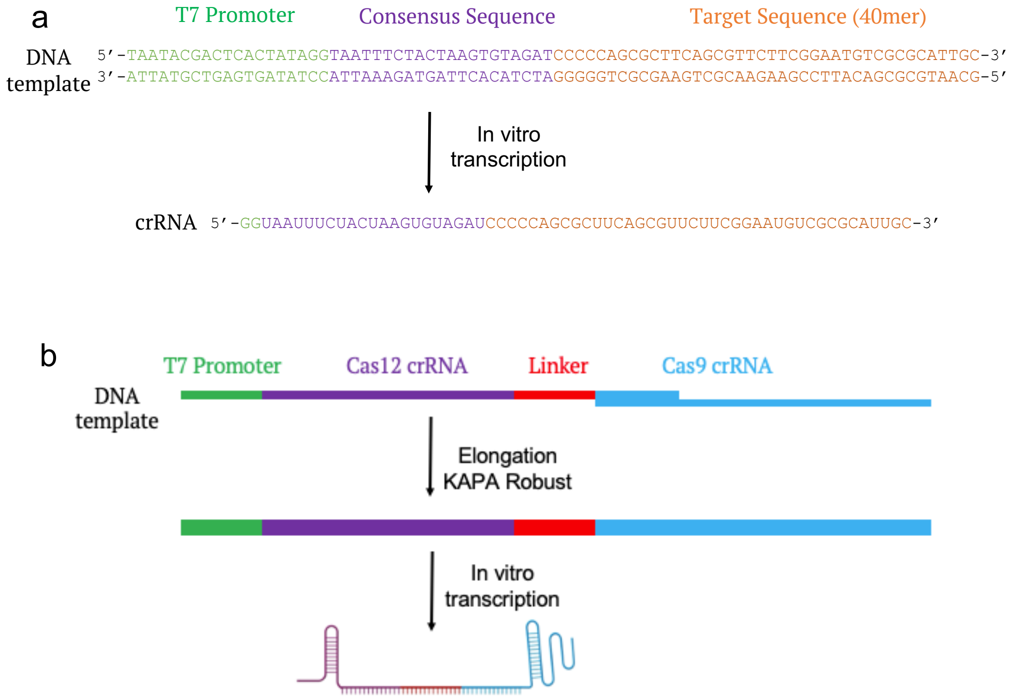

Supplementary Figure 1. Schematic showing transcription crRNAs. (a) In-vitro transcription of monovalent crRNA and (b) bivalent crRNA using HiScribe™ T7 Quick High Yield RNA Synthesis Kit. The sequences are colored according to the labeled domains.

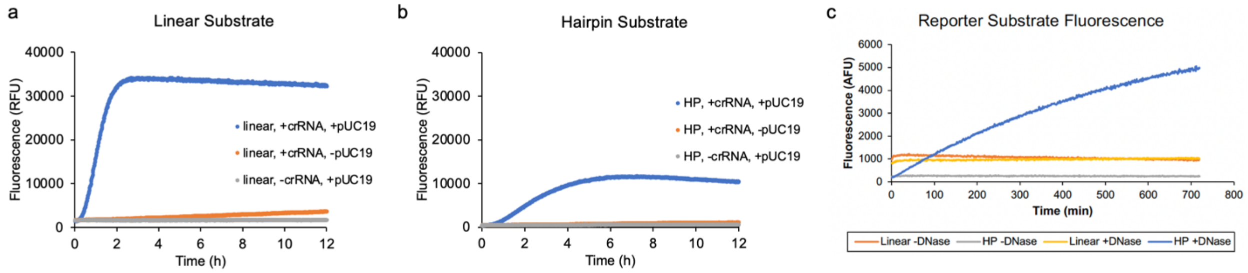

Supplementary Figure 2. Trans-cleavage activity of DNA reporter substrates. (a) Cas12 trans-cleavage activity between linear substrate and (b) hairpin substrate showed that Cas12 cleaved the linear substrate better than the hairpin substrate. (c) Shows the effect of DNase on both DNA reporter substrates with hairpin substrate having a lower background fluorescence yet increased signal in comparison to the linear substrate.

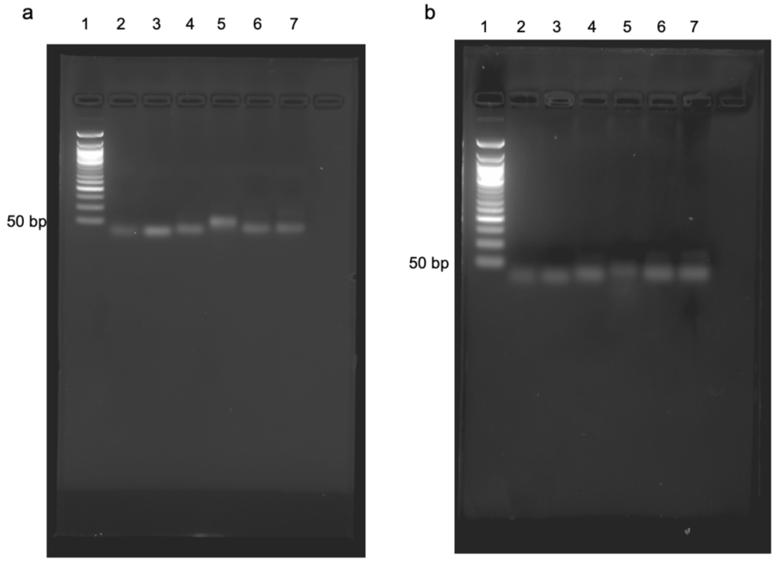

Supplementary Figure 3. Denaturation gel to confirm dimerization of crRNA-30. (a) Gel image before denaturation (b) Gel image of denatured gel. Lane 1 represents the 50 bp DNA ladder, lanes 2 – 6 represent the different length of monovalent crRNAs ranging from crRNA-15 to 40 in the order 38, 43, 48, 53, and 58 ribonucleotides respectively in (a) and (b). The gel shows a slight change in band sizes across the different crRNAs.

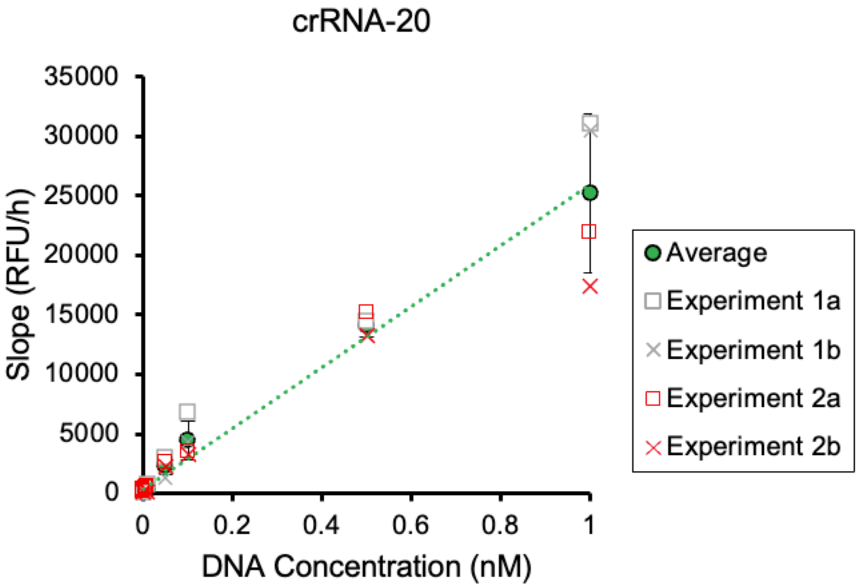

Supplementary Figure 4. Comparison of Cas12 and crRNA activity from different experiments. Data showing Cas12 activity from using the different batches of Cas12 and crRNA from two independent experiments. Standard deviation was calculated using n=4.

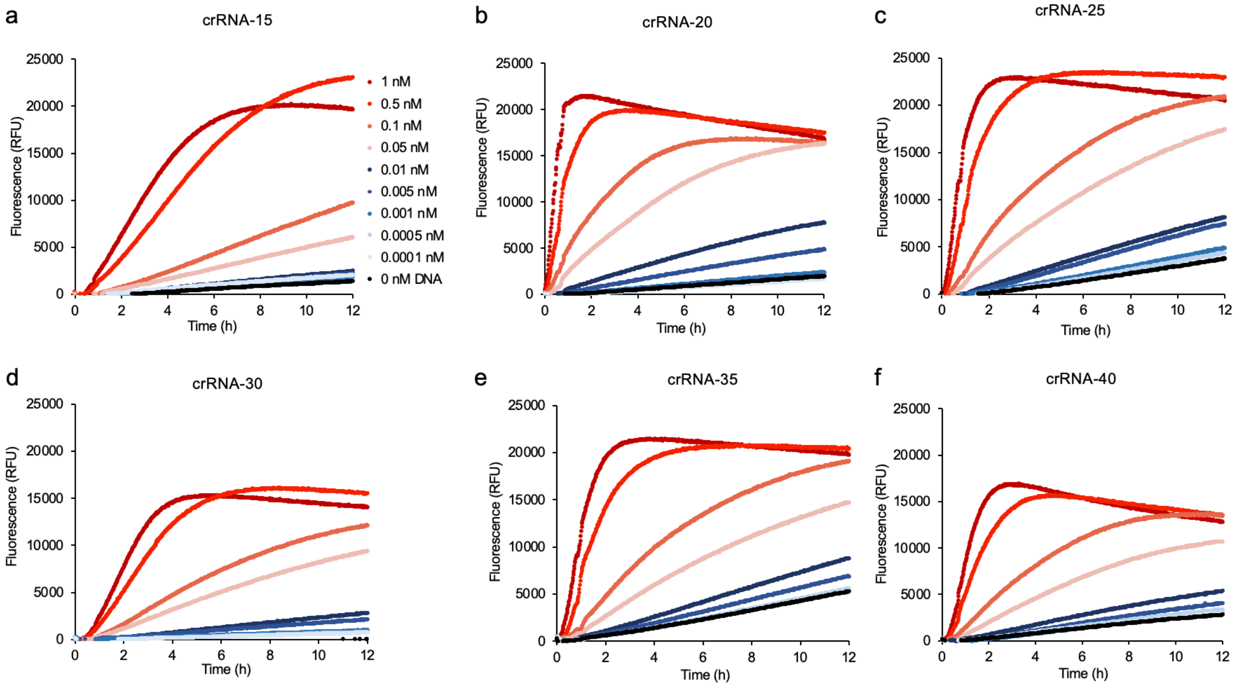

Supplementary Figure 5. LoD studies for LbCas12a. Fluorescence kinetic plots for different concentrations of target DNA for each crRNA. Experiments were run in duplicate for each length of crRNA and average results are shown (*n = 2*). Concentrations for Cas12, crRNA and reporter substrate were 200 nM, 12.5 nM and 100 nM, respectively. (a) crRNA-15 showed significant activity with Cas12 at higher concentration of target DNA. However, (b) crRNA-20 produced the fastest fluorescence signal among all crRNAs at 1 nM DNA concentration. (c) crRNA-25 showed the next highest signal at 1 nM DNA concentration. (d) The activity of Cas12 with crRNA-30 was reduced compared to (d) crRNA-35 and (e) crRNA-40. In summary, the activity of Cas12 depreciated with decreased concentration of target DNA for most DNA.

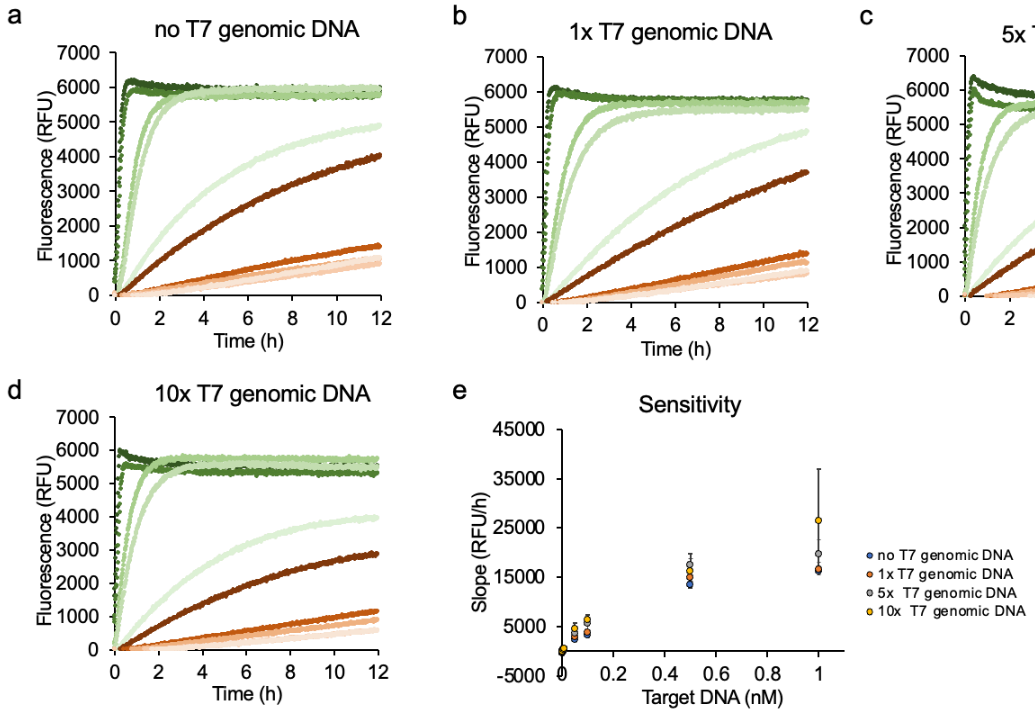

Supplementary Figure 6. Performance of Cas12-crRNA-20 and target DNA in a mixed pool of non-target DNA. Cas12 activity (a) with target DNA only, (b) with 1x T7 genomic DNA, (c) 5x T7 genomic DNA, and (d) 10x T7 genomic DNA. (e) Summary of sensitivity studies showing no significant change in Cas12 activity. Error bars show standard deviation (*n*=2).

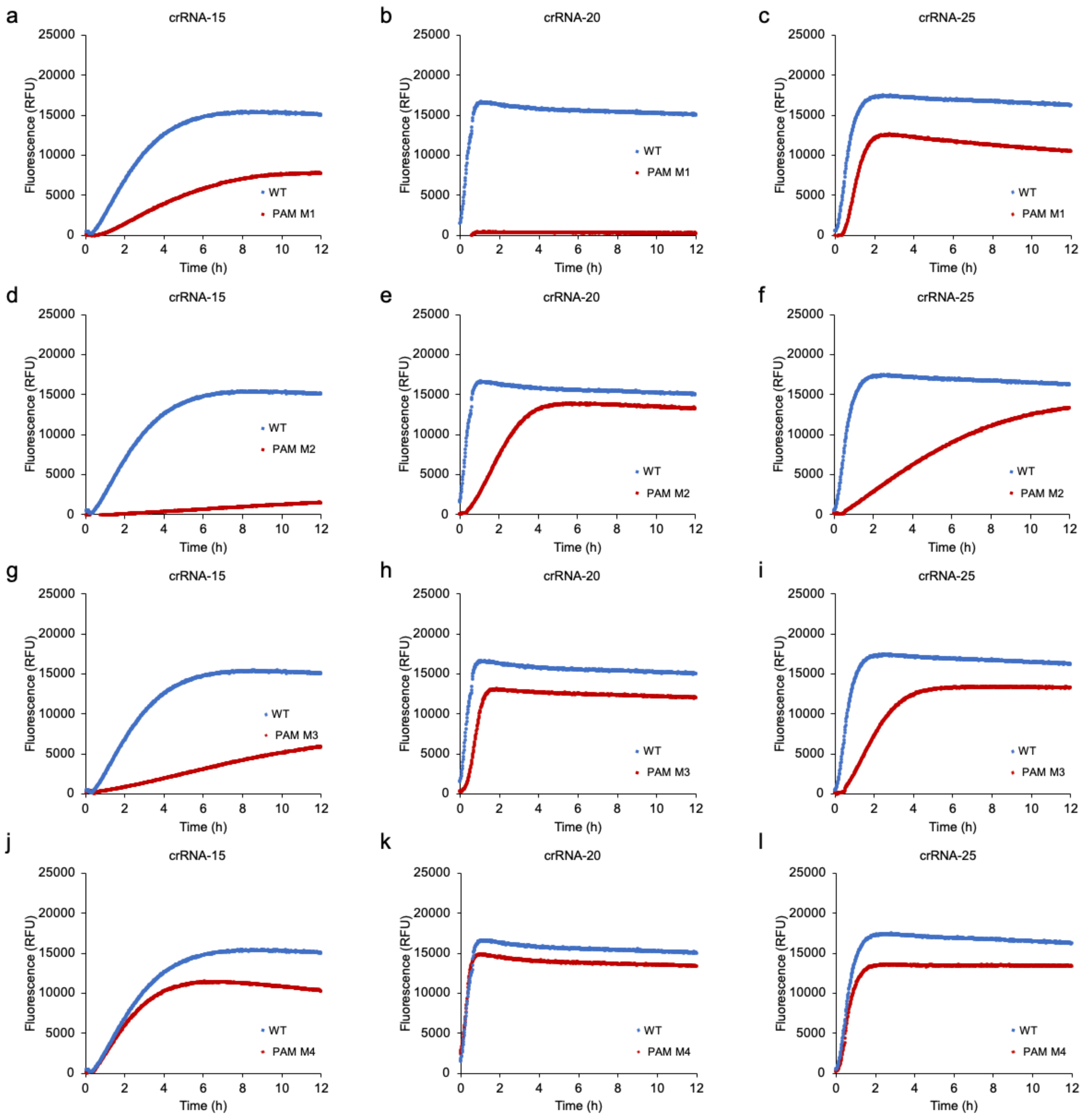
a
crRNA-15
Fluorescence (RFU)
Time (h)
WT
PAM M1
b
crRNA-20
Fluorescence (RFU)
Time (h)
WT
PAM M1
c
crRNA-25
Fluorescence (RFU)
Time (h)
WT
PAM M1
d
crRNA-15
Fluorescence (RFU)
Time (h)
WT
PAM M2
e
crRNA-20
Fluorescence (RFU)
Time (h)
WT
PAM M2
f
crRNA-25
Fluorescence (RFU)
Time (h)
WT
PAM M2
g
crRNA-15
Fluorescence (RFU)
Time (h)
WT
PAM M3
h
crRNA-20
Fluorescence (RFU)
Time (h)
WT
PAM M3
i
crRNA-25
Fluorescence (RFU)
Time (h)
WT
PAM M3
j
crRNA-15
Fluorescence (RFU)
Time (h)
WT
PAM M4
k
crRNA-20
Fluorescence (RFU)
Time (h)
WT
PAM M4
l
crRNA-25
Fluorescence (RFU)
Time (h)
WT
PAM M4

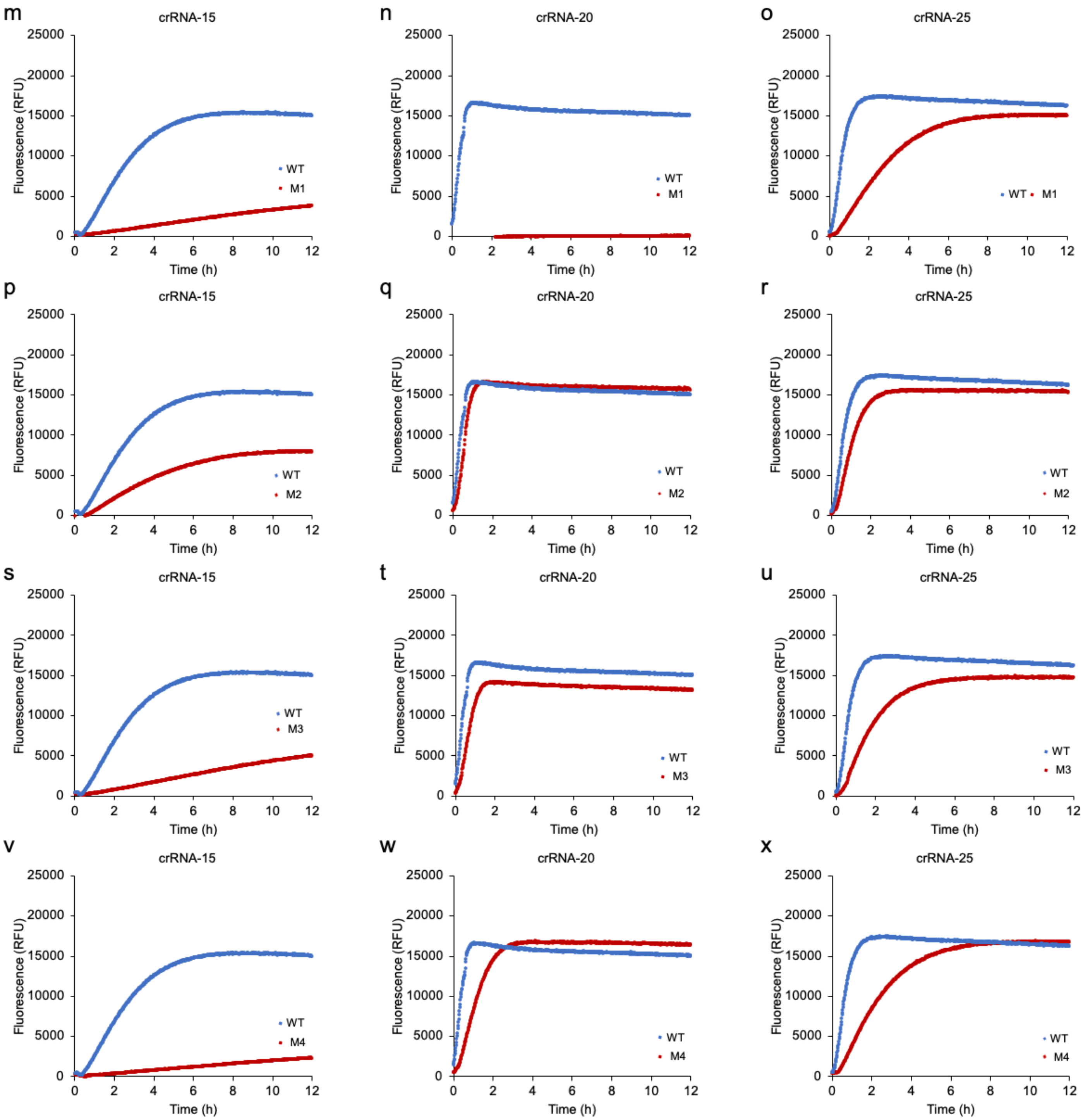

m
crRNA-15
WT
M1
n
crRNA-20
WT
M1
o
crRNA-25
WT
M1
p
crRNA-15
WT
M2
q
crRNA-20
WT
M2
r
crRNA-25
WT
M2
s
crRNA-15
WT
M3
t
crRNA-20
WT
M3
u
crRNA-25
WT
M3
v
crRNA-15
WT
M4
w
crRNA-20
WT
M4
x
crRNA-25
WT
M4
Fluorescence (RFU)
Time (h)
0
5000
10000
15000
20000
25000
0
2
4
6
8
10
12

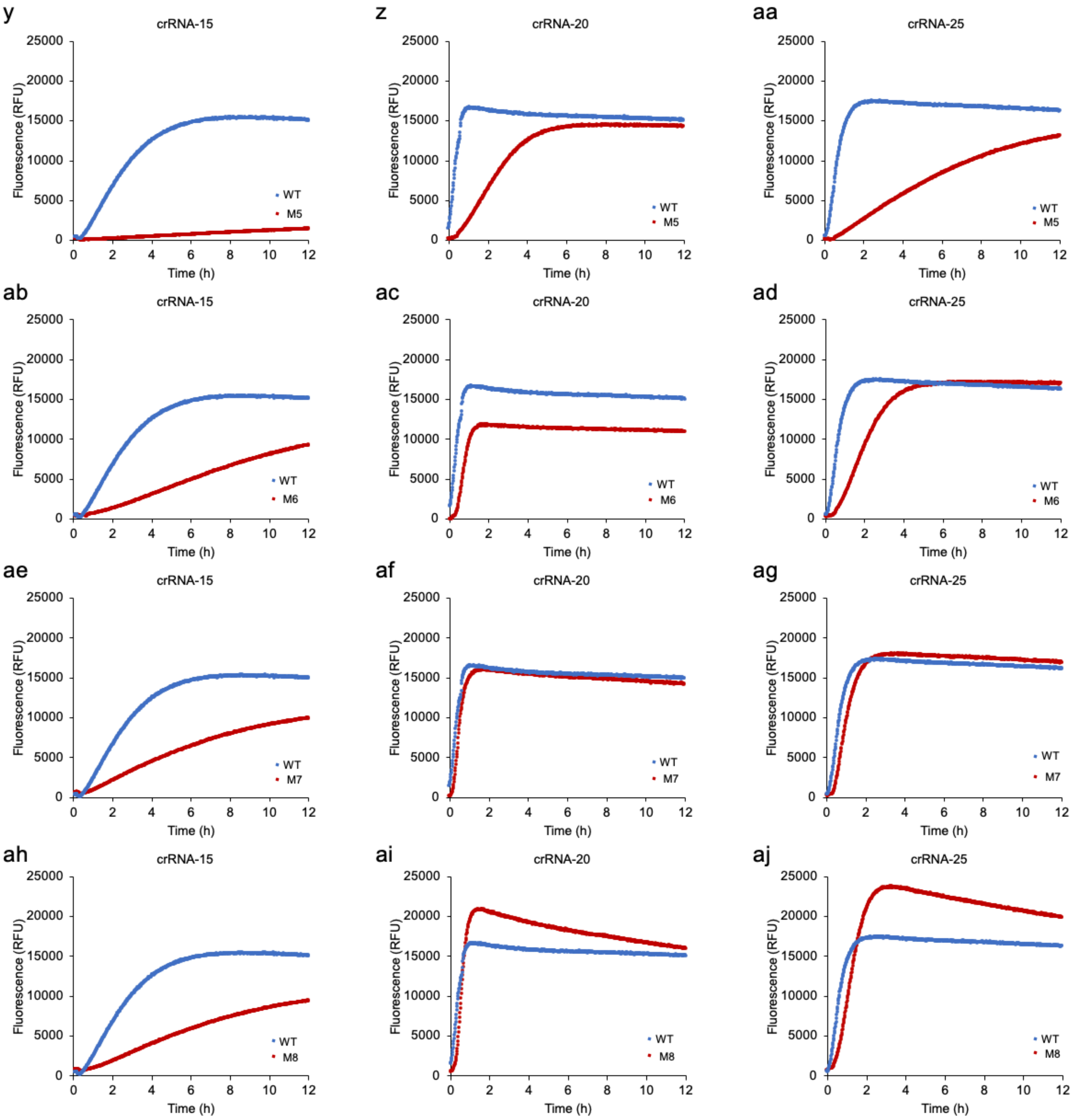
y
crRNA-15
Fluorescence (RFU)
Time (h)
WT
M5
z
crRNA-20
WT
M5
aa
crRNA-25
WT
M5
ab
crRNA-15
WT
M6
ac
crRNA-20
WT
M6
ad
crRNA-25
WT
M6
ae
crRNA-15
WT
M7
af
crRNA-20
WT
M7
ag
crRNA-25
WT
M7
ah
crRNA-15
WT
M8
ai
crRNA-20
WT
M8
aj
crRNA-25
WT
M8
0
5000
10000
15000
20000
25000
0
2
4
6
8
10
12

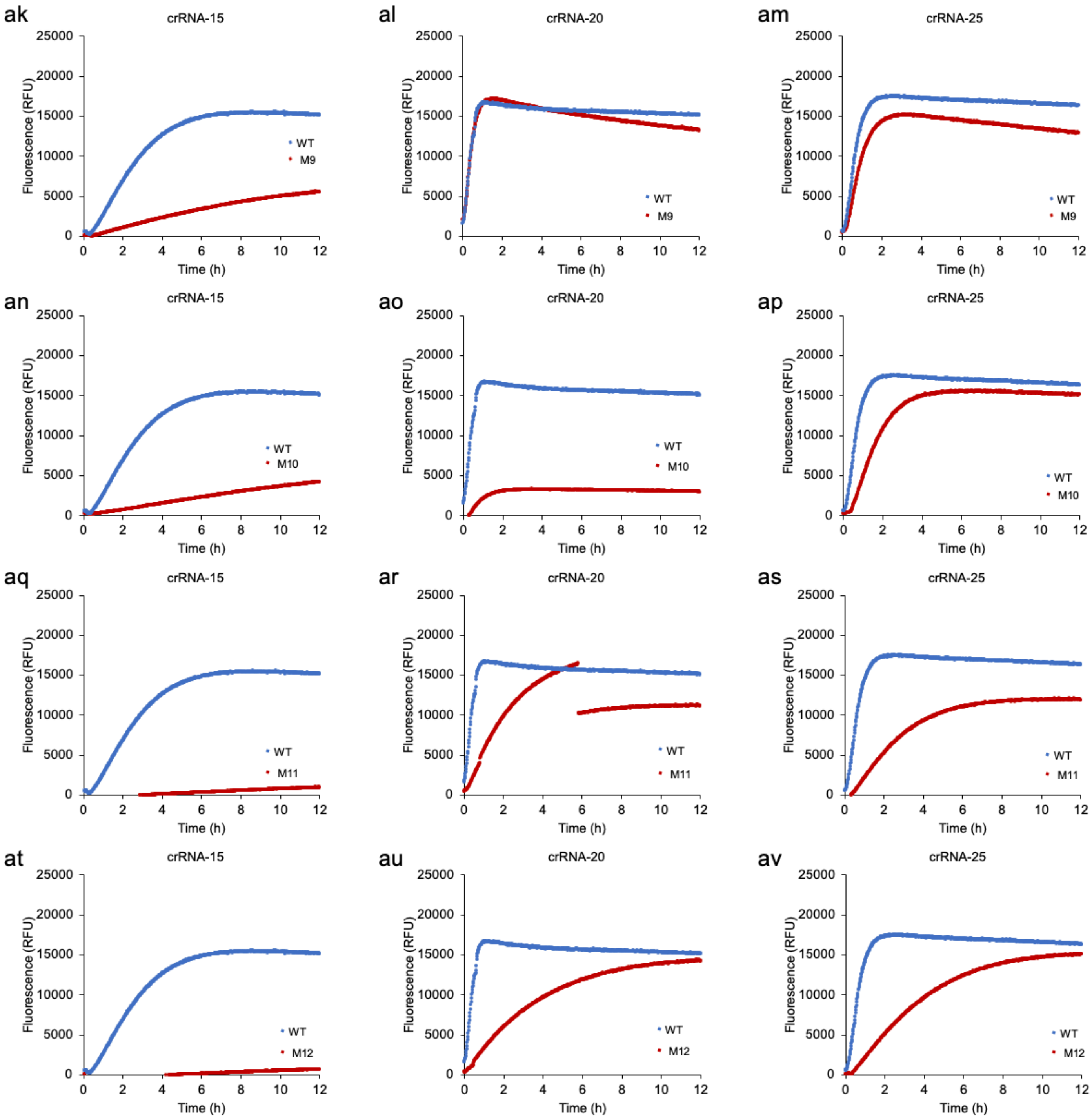
ak
crRNA-15
WT
M9
al
crRNA-20
WT
M9
am
crRNA-25
WT
M9
an
crRNA-15
WT
M10
ao
crRNA-20
WT
M10
ap
crRNA-25
WT
M10
aq
crRNA-15
WT
M11
ar
crRNA-20
WT
M11
as
crRNA-25
WT
M11
at
crRNA-15
WT
M12
au
crRNA-20
WT
M12
av
crRNA-25
WT
M12
Fluorescence (RFU)
Time (h)
0
5000
10000
15000
20000
25000
0
2
4
6
8
10
12

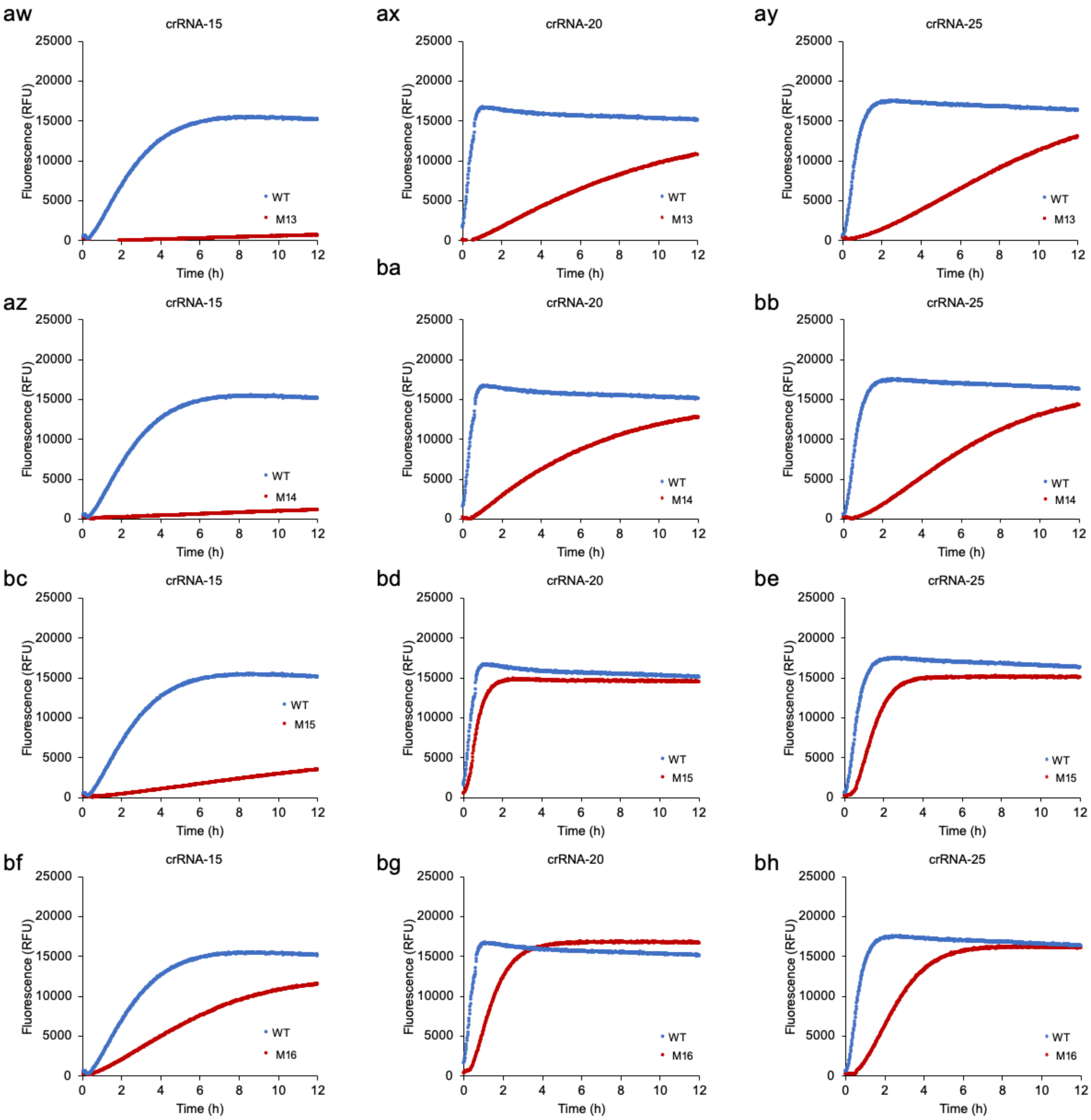
aw
crRNA-15
WT
M13
ax
crRNA-20
WT
M13
ay
crRNA-25
WT
M13
az
crRNA-15
WT
M14
ba
crRNA-20
WT
M14
bb
crRNA-25
WT
M14
bc
crRNA-15
WT
M15
bd
crRNA-20
WT
M15
be
crRNA-25
WT
M15
bf
crRNA-15
WT
M16
bg
crRNA-20
WT
M16
bh
crRNA-25
WT
M16
Fluorescence (RFU)
Time (h)
0
5000
10000
15000
20000
25000
0
2
4
6
8
10
12

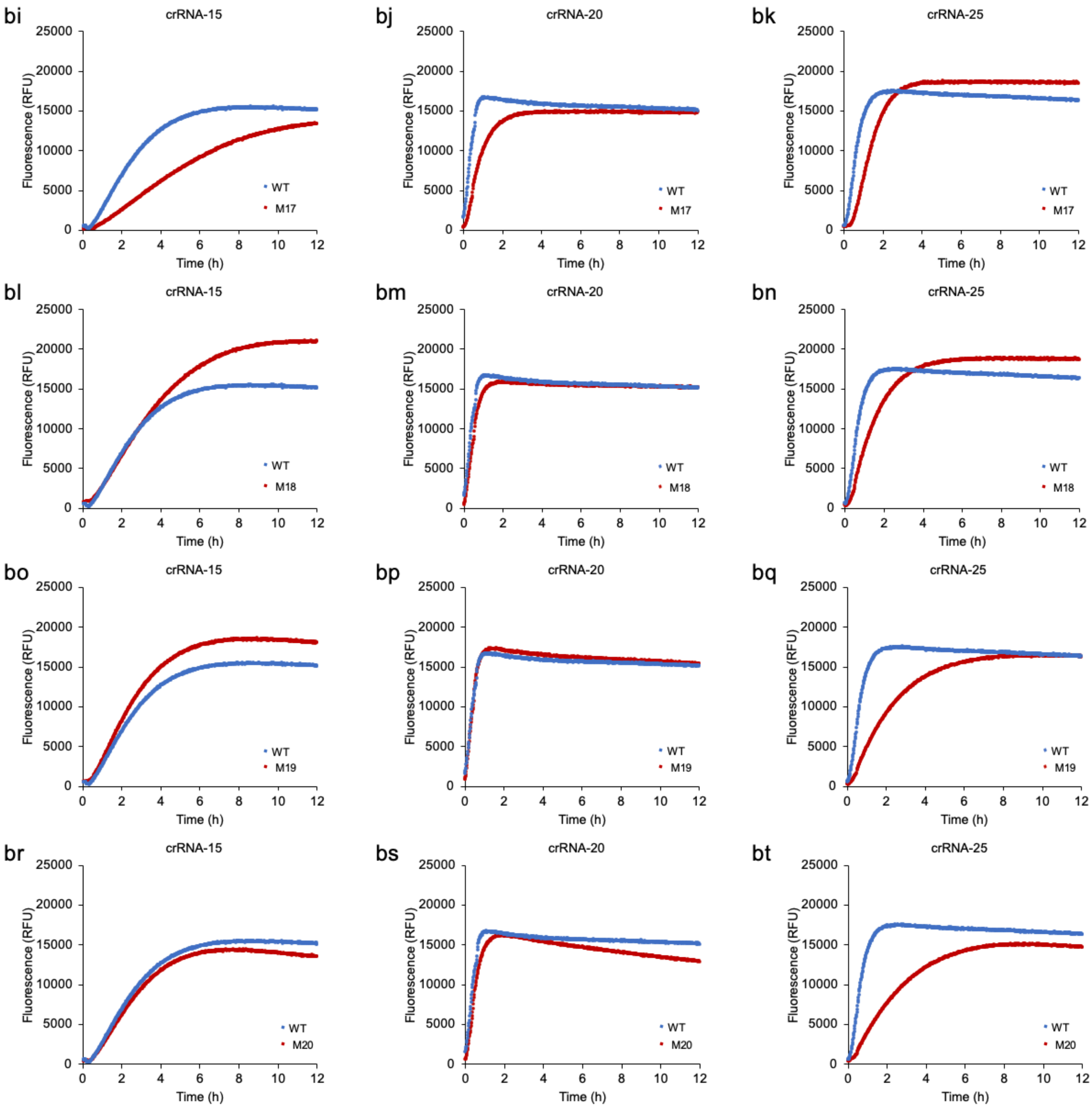
bi
crRNA-15
bj
crRNA-20
bk
crRNA-25
bl
crRNA-15
bm
crRNA-20
bn
crRNA-25
bo
crRNA-15
bp
crRNA-20
bq
crRNA-25
br
crRNA-15
bs
crRNA-20
bt
crRNA-25
Fluorescence (RFU)
Time (h)
WT
M17
M18
M19
M20
0
5000
10000
15000
20000
25000
0 2 4 6 8 10 12

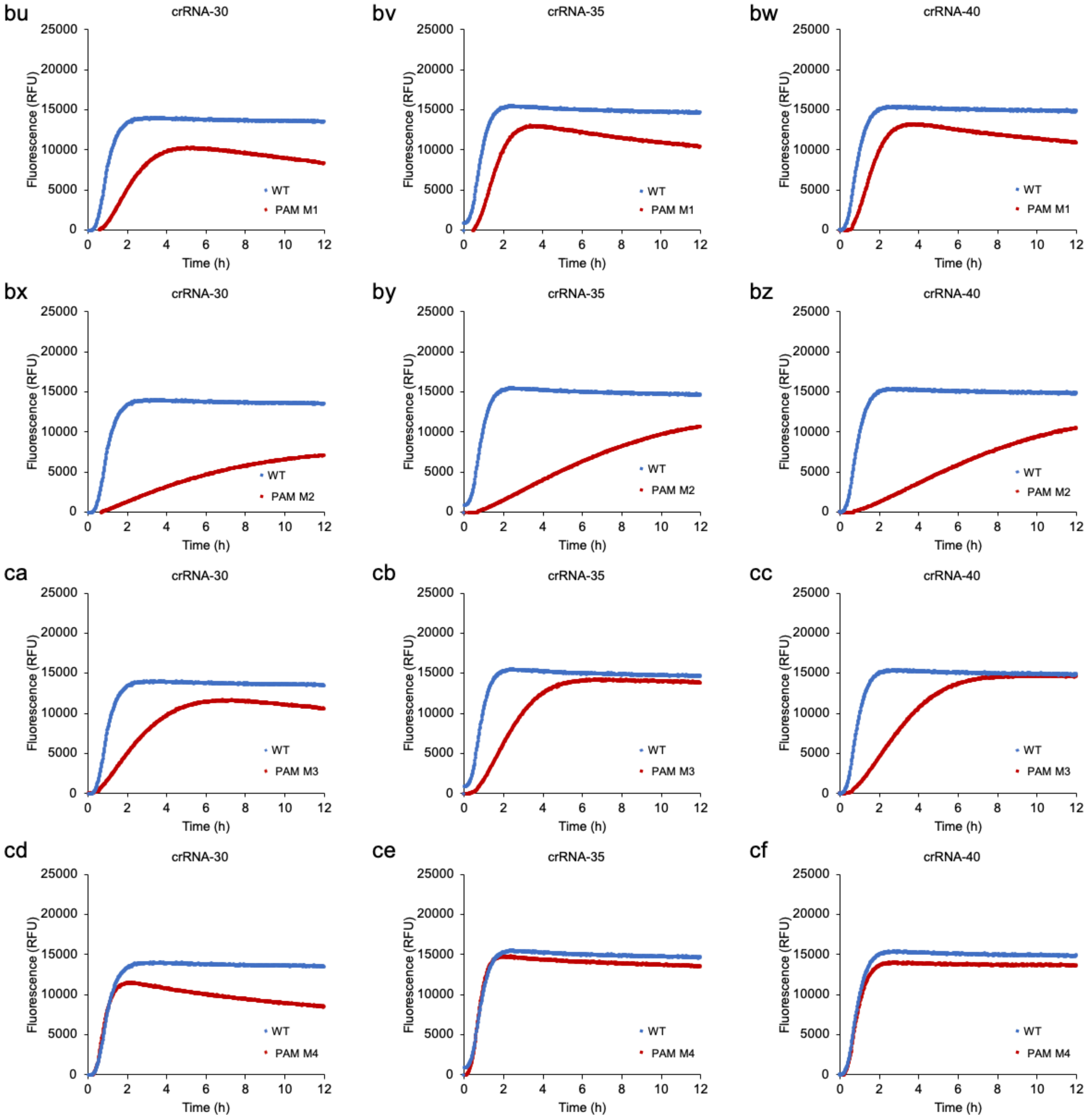
bu
crRNA-30
WT
PAM M1
bv
crRNA-35
WT
PAM M1
bw
crRNA-40
WT
PAM M1
bx
crRNA-30
WT
PAM M2
by
crRNA-35
WT
PAM M2
bz
crRNA-40
WT
PAM M2
ca
crRNA-30
WT
PAM M3
cb
crRNA-35
WT
PAM M3
cc
crRNA-40
WT
PAM M3
cd
crRNA-30
WT
PAM M4
ce
crRNA-35
WT
PAM M4
cf
crRNA-40
WT
PAM M4
Fluorescence (RFU)
Time (h)
0
5000
10000
15000
20000
25000
0 2 4 6 8 10 12

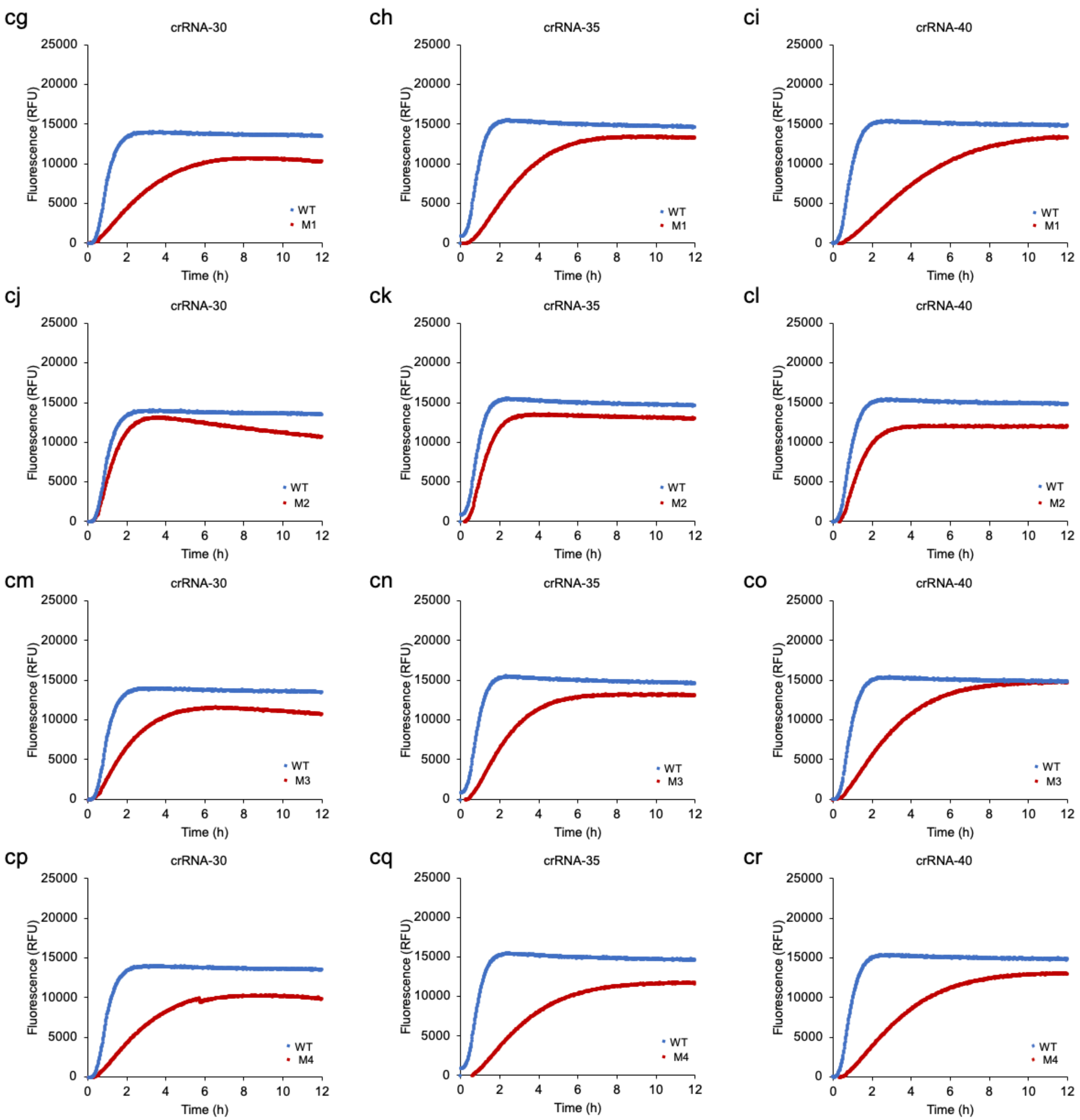
cg
crRNA-30
ch
crRNA-35
ci
crRNA-40
cj
crRNA-30
ck
crRNA-35
cl
crRNA-40
cm
crRNA-30
cn
crRNA-35
co
crRNA-40
cp
crRNA-30
cq
crRNA-35
cr
crRNA-40
Fluorescence (RFU)
0
5000
10000
15000
20000
25000
Time (h)
0
2
4
6
8
10
12
WT
M1
M2
M3
M4

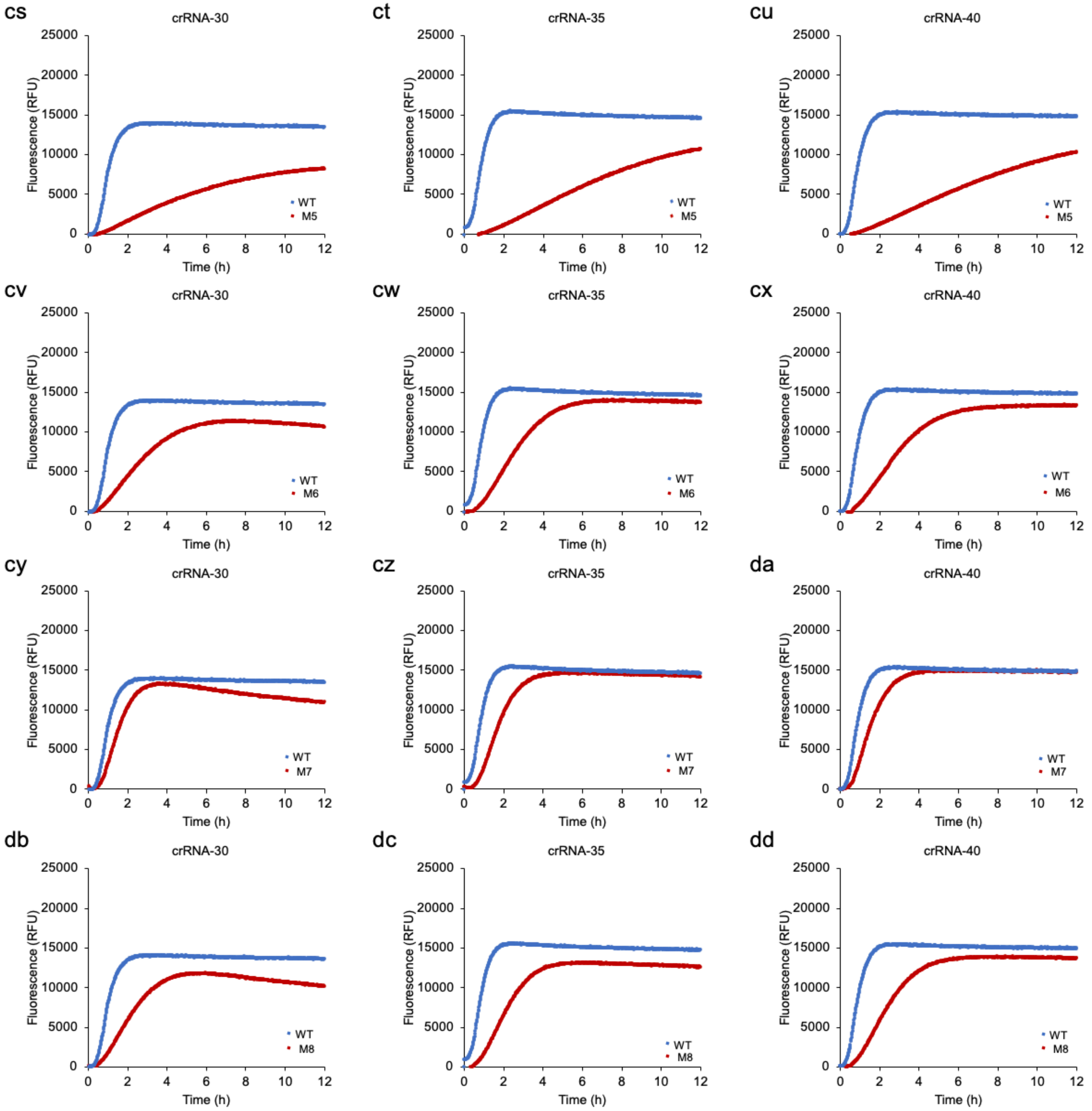
cs
crRNA-30
ct
crRNA-35
cu
crRNA-40
WT
M5
cv
crRNA-30
cw
crRNA-35
cx
crRNA-40
WT
M6
cy
crRNA-30
cz
crRNA-35
da
crRNA-40
WT
M7
db
crRNA-30
dc
crRNA-35
dd
crRNA-40
WT
M8
Fluorescence (RFU)
Time (h)
0
5000
10000
15000
20000
25000
0
2
4
6
8
10
12

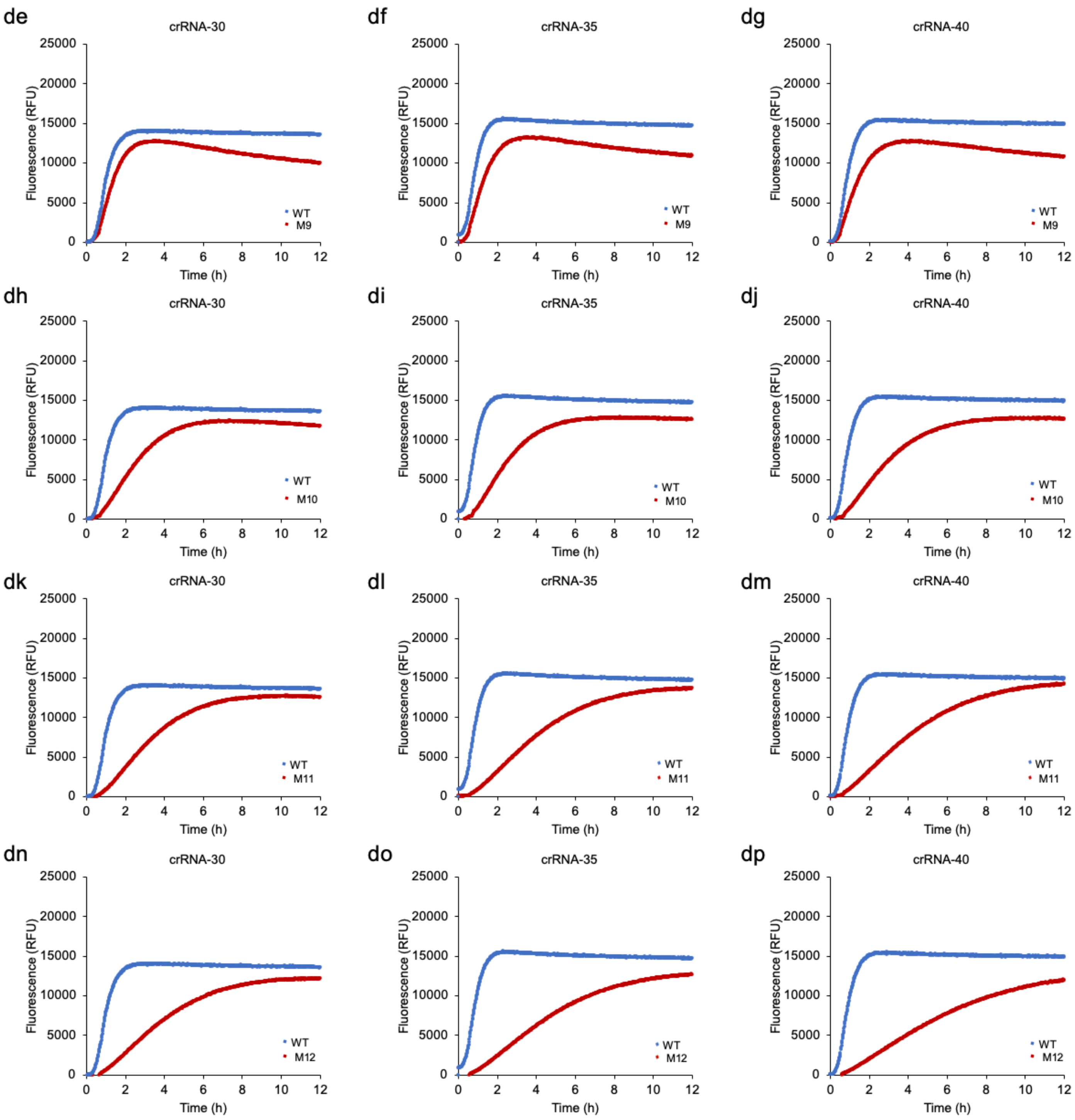
de
crRNA-30
df
crRNA-35
dg
crRNA-40
dh
crRNA-30
di
crRNA-35
dj
crRNA-40
dk
crRNA-30
dl
crRNA-35
dm
crRNA-40
dn
crRNA-30
do
crRNA-35
dp
crRNA-40
Fluorescence (RFU)
Time (h)
WT
M9
M10
M11
M12

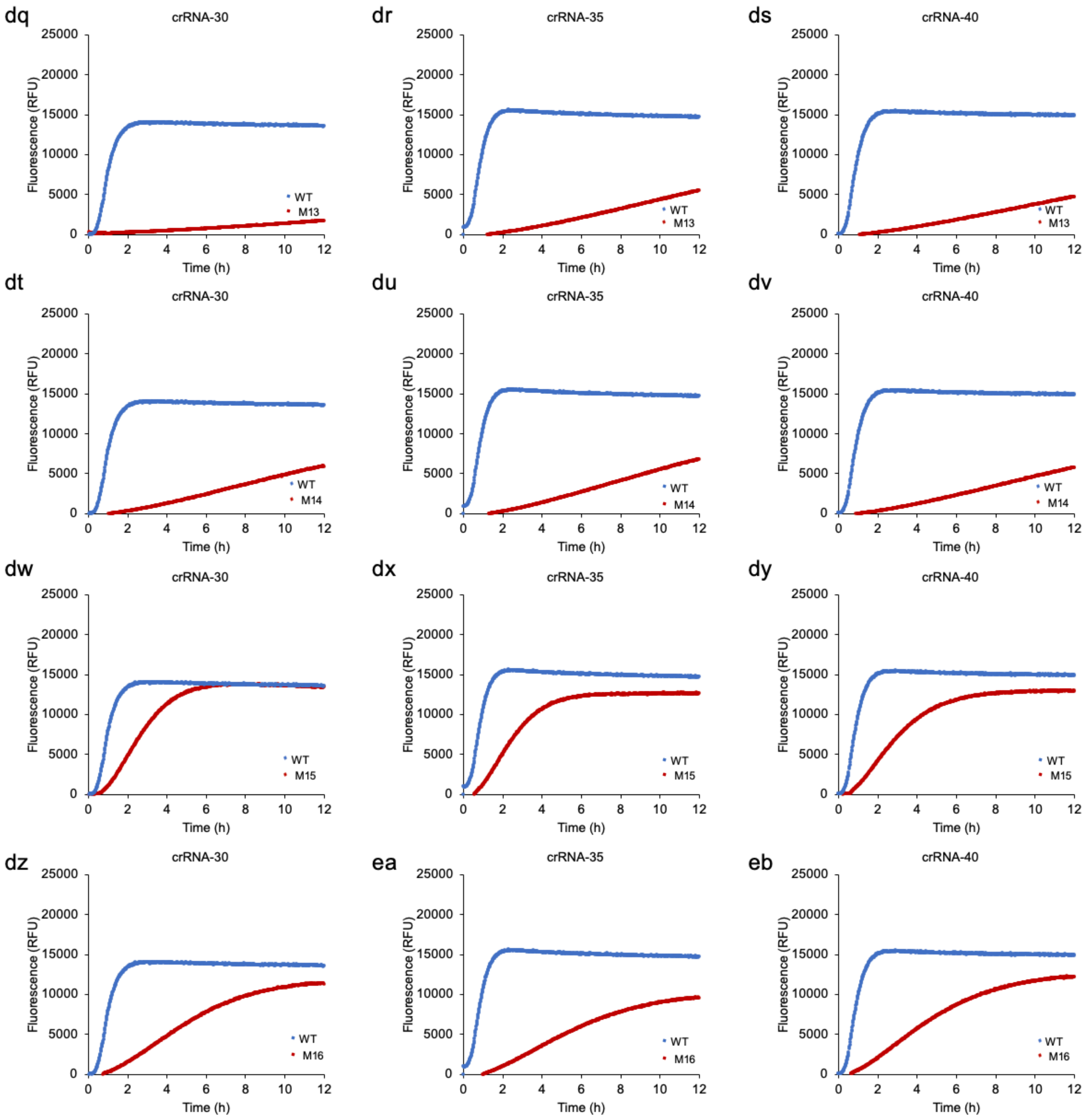

dq
crRNA-30
dr
crRNA-35
ds
crRNA-40
dt
crRNA-30
du
crRNA-35
dv
crRNA-40
dw
crRNA-30
dx
crRNA-35
dy
crRNA-40
dz
crRNA-30
ea
crRNA-35
eb
crRNA-40
Fluorescence (RFU)
Time (h)
WT
M13
M14
M15
M16

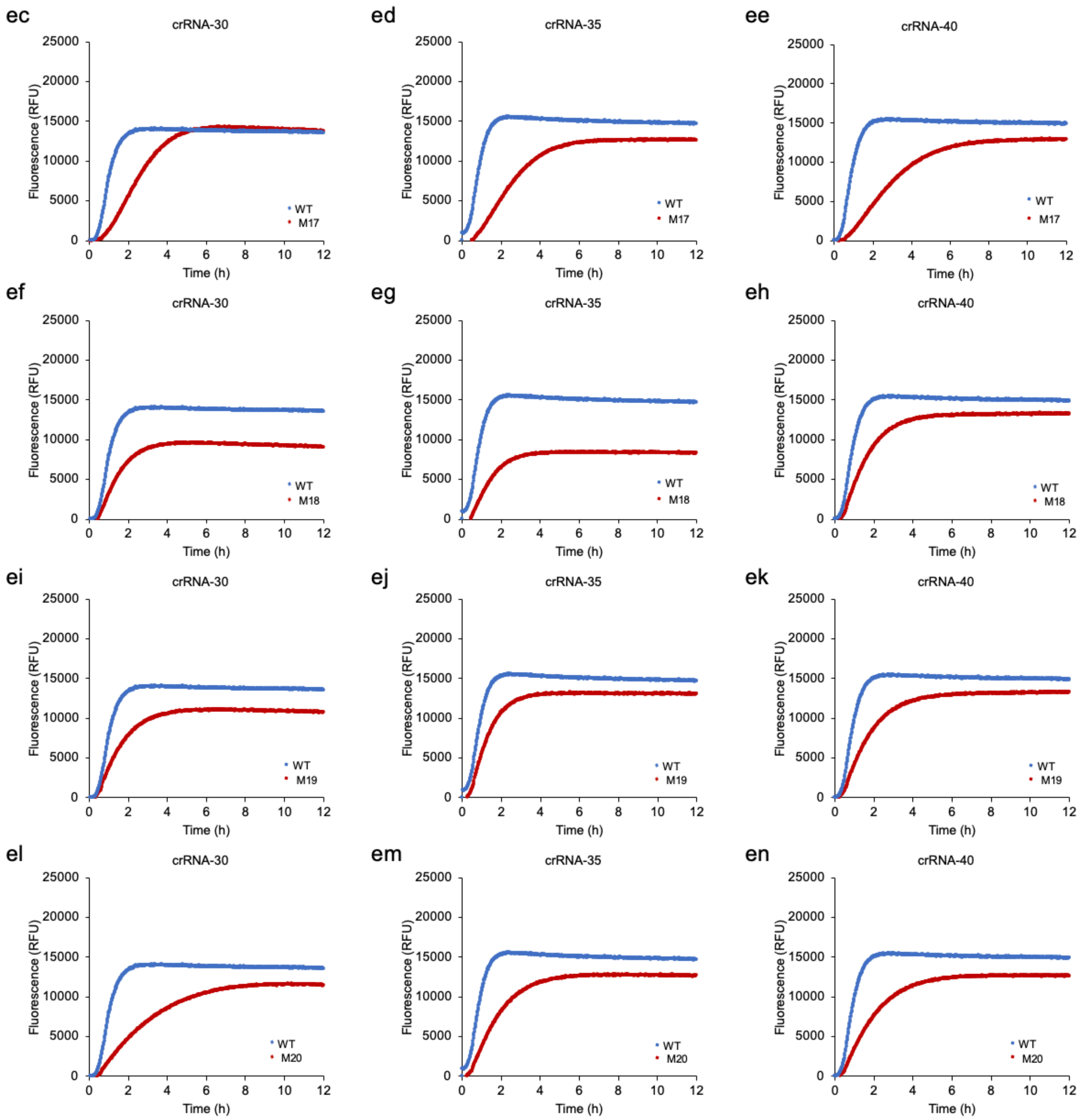

Supplementary Figure 7. SNP studies for Cas12. Fluorescence kinetic assay of Cas12 activity comparing WT in blue to SNPs in red for all lengths of crRNA.

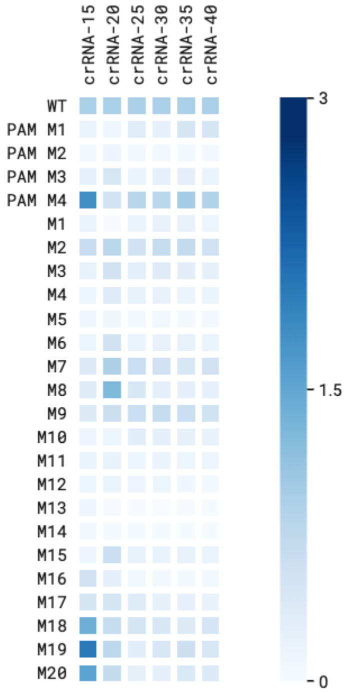

Supplementary Figure 8. Heat map for Cas12 SNP studies. SNP mutation intensities were normalized to 1 for wildtype with colors ranging from light blue to deep blue.

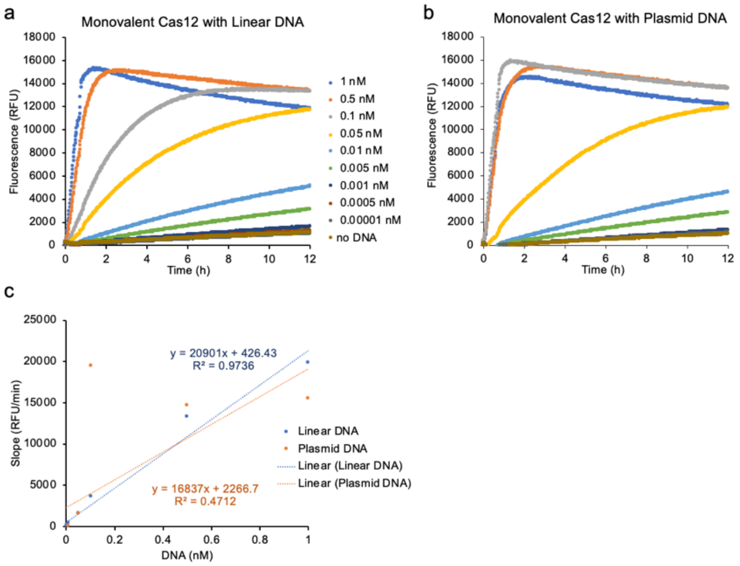

Supplementary Figure 9. Comparison of Cas12 activity for linear and plasmid DNA. (a) Trans-cleavage activity of Cas12 with different concentrations of DNA. The experiments were run in duplicate and average data is shown (*n = 2)*. Concentrations for Cas12, crRNA-20 and reporter substrate were 200 nM, 12.5 nM and 100 nM, respectively. The results show that the 1 nM and 0.5 nM linear DNA have similar maximum velocity. (b) Similar to (a) other than plasmid DNA was used as activator DNA in this experiment. Here, we see that the 1 nM, 0.5 nM and 0.1 nM produced similar activity. (c) A plot showing Cas12 sensitivity to both linear and plasmid DNA.

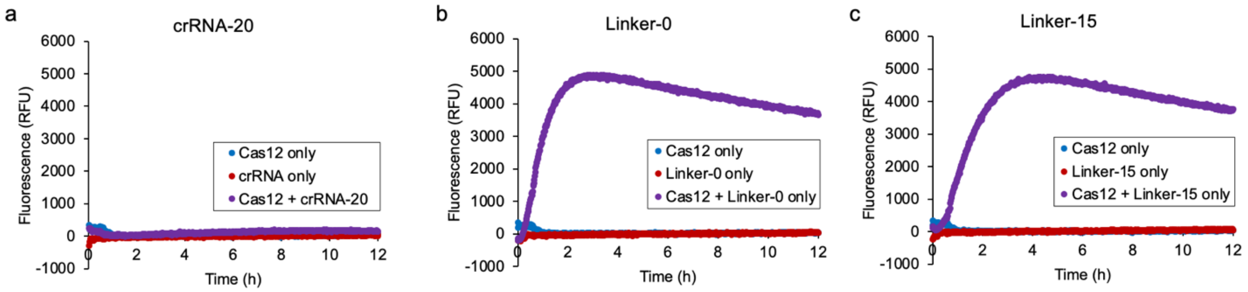

Supplementary Figure 10. Investigating background activity with no DNA. (a) Cas12 activity with and without crRNA shows no background activity in the absence of target DNA. (b) Cas12 activity with and without bivalent crRNA, linker-0. The purple curve shows a high background activity without DNA which is the same as what we see in (c) with linker-15.

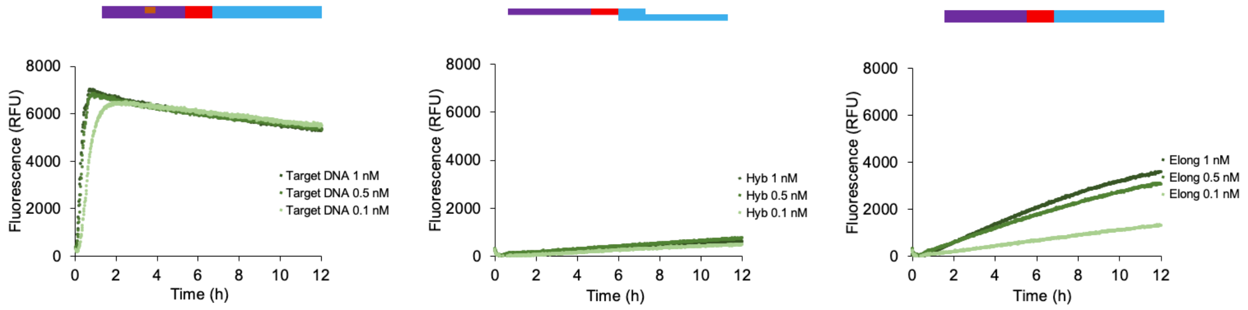

Supplementary Figure 11. Investigating background activity with different DNA. (a) fully complementary target DNA, (b) hybridized DNA used for making bivalent crRNA template for transcription, and (c) fully extended DNA used for bivalent crRNA transcription (orange is PAM, purple is Cas12 crRNA, red is linker, and blue is Cas9 crRNA. Though activity is observed for the extended DNA, it is not as much as is observed in the bivalent crRNA only (Figure S8).

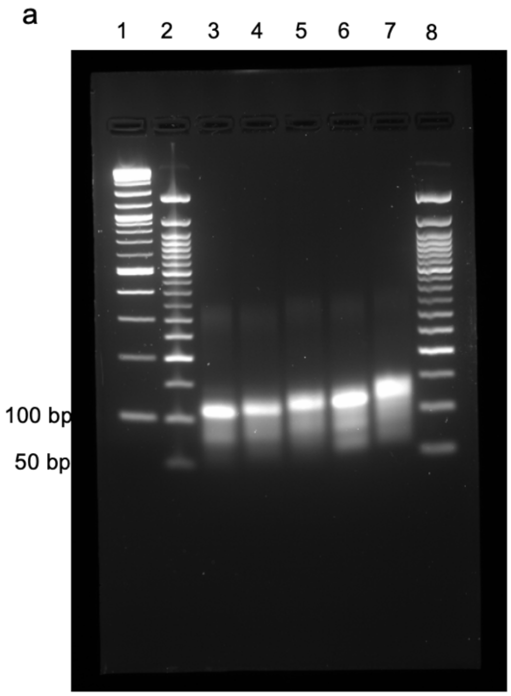

Supplementary Figure 12. Uncropped gel image from Figure 6a. (a) Gel electrophoresis characterization of bivalent crRNAs. Lane 1, 2 and 8 represents the 100 bp, 50 bp and 50 bp DNA ladder respectively, lanes 3 – 7 represent the different length of bivalent crRNA in the order 142, 147, 152, 157, and 162 ribonucleotides respectively. The gel shows a progressive upward shift as the spacer length of bivalent crRNA increases.

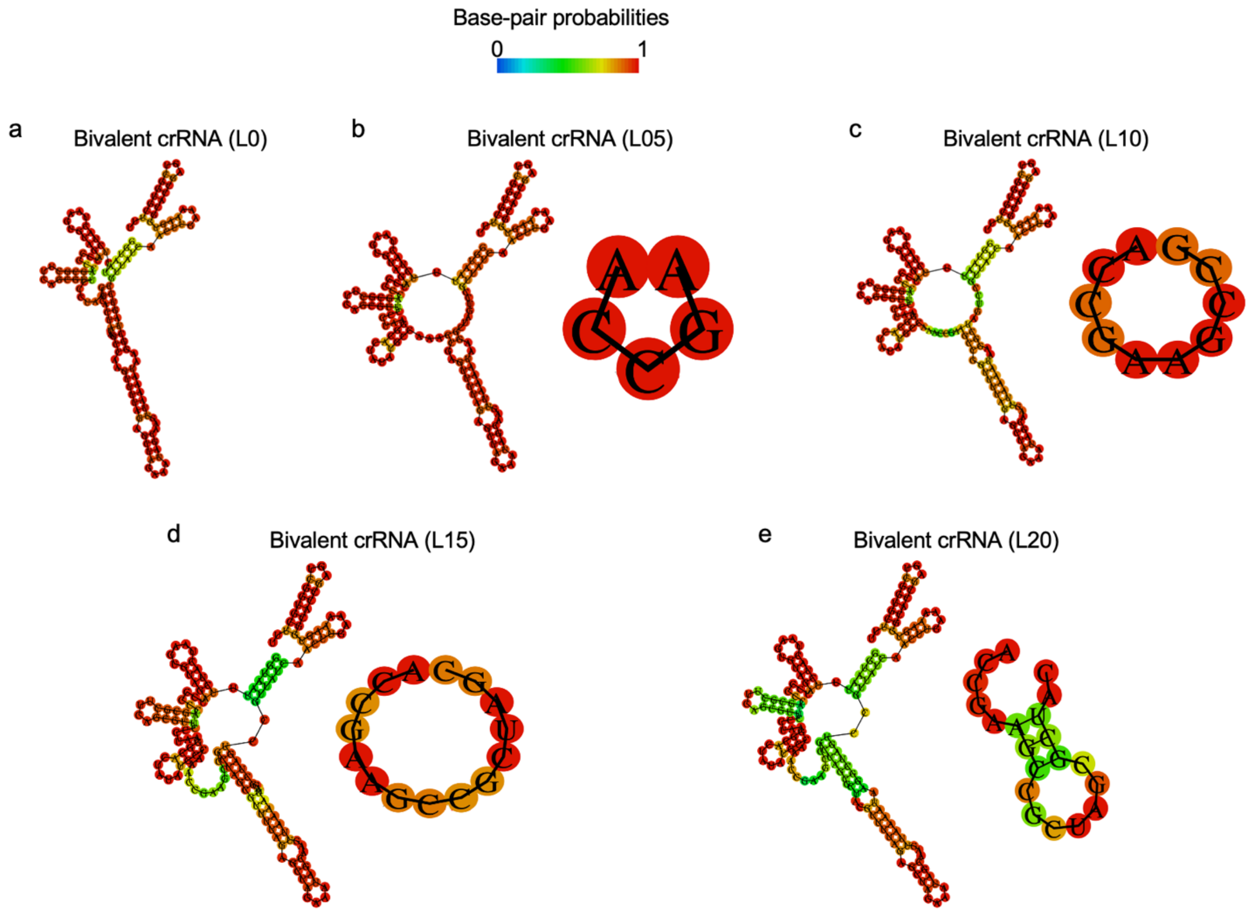

Supplementary Figure 13. Predicted bivalent crRNA secondary structures. (a) Secondary structure of bivalent crRNA with no linker or L0. (b) Secondary structure of bivalent crRNA with L5. (c) Secondary structure of bivalent crRNA with L10. (d) Secondary structure of bivalent crRNA with L15. (e) Secondary structure of bivalent crRNA with L20. RNA structures were generated using RNAfold webserver.
